# Astaxanthin suppresses the malignant behaviors of nasopharyngeal carcinoma cells by blocking PI3K/AKT and NF-κB pathways via miR-29a-3p

**DOI:** 10.1186/s41021-024-00304-w

**Published:** 2024-04-22

**Authors:** Yajia Xu, Chengyi Jiang

**Affiliations:** Department of Otolaryngology, Head and Neck Surgery, The First Affiliated Hospital of Bengbu Medical College, No.287 Changhuai Road, 233000 Bengbu, Anhui China

**Keywords:** Astaxanthin, Nasopharyngeal carcinoma, C666-1 cells, PI3K/AKT, NF-κB

## Abstract

**Background:**

As a natural extraction, astaxanthin is gaining increasing attention because of its safety and anti-tumor properties. It has been reported to participate in the progression of various types of cancer such as gastric cancer and ovarian cancer. Nevertheless, the role of astaxanthin in nasopharyngeal carcinoma (NPC) has not been investigated.

**Object:**

The study aimed to explore the anticancer mechanism of astaxanthin in regulating NPC cell proliferation, cell cycle progression, apoptosis, migration, and invasion.

**Methods:**

Human NPC cells (C666-1) were treated with different concentrations of astaxanthin (0, 1, 10, 20 mg/mL) followed by detection of cell viability. Then, C666-1 cell proliferation, apoptosis, cell cycle progression, invasion, and migration in response to 10 mg/mL astaxanthin, LY294002 (PI3K/AKT inhibitor) or parthenolide (PTL; NF-κB inhibitor) treatment were measured using cell counting kit-8 assay, colony forming assay, flow cytometry analyses, Transwell assay, and wound healing assay, respectively. Western blotting was performed to quantify protein levels of factors involved in PI3K/AKT and NF-κB signaling pathways, cell cycle phase markers (Cyclin D1, p21) and apoptotic markers (Bcl-2 and Bax).

**Results:**

C666-1 cell proliferation, invasion, and migration were significantly suppressed by astaxanthin while cell apoptosis and cell cycle arrest at G1 phase were effectively enhanced in the context of 10 mg/mL astaxanthin. Protein levels of p-AKT, p-P65 and p-IκB levels were suppressed by astaxanthin treatment. After LY294002 or PTL treatment, the suppressive impact of astaxanthin on C666-1 cell process was strengthened, accompanied by the more obvious decrease in cell activity and cell colony number, more enhanced cell apoptosis and G1 phase arrest, and further inhibited cell migration and invasion. Moreover, the inhibitory effect of astaxanthin on Cyclin D1 and Bcl-2 protein levels as well as the promoting impact of astaxanthin on p21 and Bax were also amplified in combination with LY294002 or PTL treatment.

**Conclusions:**

Astaxanthin significantly suppresses NPC cell proliferation, cell cycle arrest, migration, invasion while promoting cell apoptosis by inactivating PI3K/AKT and NF-κB pathways. The study first reveals the anticancer role of astaxanthin in NPC, providing a potential candidate for NPC treatment.

## Introduction

Nasopharyngeal carcinoma (NPC) is a malignant epithelial tumor in the nasopharynx with a high incidence in North Africa and Southeast Asia [1, 2]. Infection of Epstein-Barr virus is a leading trigger of NPCand the infection is implicated in NPC cell proliferation, migration, invasion, and tumor immune escape through virus-encoded proteins or noncoding RNAs [3]. Other risk factors, such as genetic predisposition and environmental factors can also induce NPC [4, 5]. Nowadays, the only curative treatment for NPC is radiotherapy [6]. In addition, immune therapy and targeted therapy are promising options for NPC metastasis and recurrence [6]. NPC is often diagnosed at a late stage and is related to poor prognosis (five-year survival rate: 63%) [7]. Finding more effective treatment is extremely important to improve the prognosis of patients with NPC.

Astaxanthin is a reddish-orange pigment and a liposoluble carotenoid extracted from alga *Haematococcus pluvialis*, which has been reported to have anti-inflammation and antioxidant activities and exert a crucial role in cell membranes and circulating lipoproteins [8–10]. Astaxanthin is the final form of carotenoid synthesis and has a strong ability to quench singlet oxygen and clear free radicals. To be specific, the ionone rings of the polar zone is responsible for quenching singlet oxygen, and the polyene chain of the nonpolar zone creates more antioxidant dimensions to eliminate high-energy electrons from free radicals. The unique structure allows astaxanthin to exist in cell membrane, thereby acting both inside and outside the cell, and to have strong antioxidant capacity [11, 12]. Astaxanthin serves as an anticancer agent and its anticancer activity has been validated in many types of cancer [8]. As to its anticancer role, a previous study showed that astaxanthin inhibited the proliferation of prostate cancer cells DU145 by downregulating STAT3 expression [11]. In addition, astaxanthin hampers oral cancer cell growth, invasion, and angiogenesis by abrogating PI3K/NF-κB/STAT3 signaling pathway [13]. The anti-tumor role of astaxanthin has also been reported in breast cancer [14], colon cancer [15], gastric cancer [16] and many other types of cancer. However, there are no articles revealing the function and mechanisms of astaxanthin in NPC. The novelty of this study is that the effect of astaxanthin on malignant behaviors of NPC cells was first explored.

PI3K and NF-κB signaling pathway are associated with tumorigenesis and malignant behavior of cancer cells [17, 18]. PI3K/Akt signaling controls hallmarks of cancer, such as cell survival, metastasis, and metabolism [19]. The signaling reduces the expression of proapoptotic proteins, thereby hampering tissue apoptosis and increasing cancer cell survival. Moreover, the PI3K/Akt pathway also participates in tumor environment and contributes to angiogenesis and inflammatory factor recruitment [19]. NF-κB is a proinflammatory transcription factor regulating numerous pathways to affect cancer progression, metastasis, and drug resistance [20]. In the canonical NF-κB pathway, the inhibitory IκB proteins are phosphorylated and thus liberate the κB transcription factor to translocate to nucleus and activate the target genes [21].

In this study, the present study explored the effects of astaxanthin on NPC cell proliferation, cell cycle progression, apoptosis, migration, and invasion. In addition, LY294002 (PI3K/AKT inhibitor) or parthenolide (PTL, NF-κB inhibitor) were used to explore whether astaxanthin could suppress malignant behaviors of NPCs by controlling PI3K/AKT and NF-κB pathways. The study first reveals the role of astaxanthin in NPC and provides more evidence for the anti-tumor activity of astaxanthin in different types of cancer, which might be beneficial for improving the outcome of patients with NPC.

## Materials and methods

***Cell culture*** Human NPC cell line C666-1 was purchased from ATCC (Manassas, USA). C666-1 cells were grown in Roswell Park Memorial Institute (RPMI) 1640 medium (Gibco, USA) supplemented with 10% fetal bovine saline, 1% penicillin/streptomycin at 37 °C with 5% CO_2_. C666-1 cell line has been widely used to conduct functional experiments in NPC according to previous studies [22–24].

***Cell treatment*** Astaxanthin (Sigma-Aldrich, St. Louis, USA) at concentrations of 1, 10, or 20 mg/mL astaxanthin were used to treat C666-1 cells for 24 h.

PTL (Selleckchem, Houston, USA) was dissolved in 0.05% dimethyl sulfoxide and diluted with phosphate buffered saline (Beyotime, Shanghai, China) to 1 µM concentration. C666-1 cells were treated with 1 µM PTL for 24 h.

LY294002 (MedChem Express, Monmouth Junction, NJ, USA) at the concentrations of 20 µM was used to treat C666-1 cells for 24 h.

***Detection of cell viability*** Cell counting kit-8 (CCK-8, Beyotime, China) was applied to assess C666-1 cell viability. Cells were implanted into 96-well plates (2 × 10^3^/well) and cultured for 24 h. Next, CCK-8 reagent was added into each well for 1.5 h of cell incubation. Finally, the optical density values at the wavelength of 450 nm were examined using a microplate reader (Biotek, Winooski, USA).

***Plate clone forming assay*** After implanted into 6-well plates, cells (1 × 10^3^/plate) with indicated treatment were subjected to incubation for 2 weeks. Next, the cells were rinsed with phosphate buffered saline and fixed in methanol for 15 min. After that, the cells were stained with crystal violet (Sigma-Aldrich, St. Louis, USA) for 15 min. Images were captured using Amersham Imager (GE Healthcare, Little Chalfont, UK). The number of colonies containing more than 50 cells was counted.

***Cell apoptosis detection*** C666-1 cells with indicated treatment were centrifugated at 1000 r/min for 3 min and then re-suspended in 800 µL of phosphate-buffer saline (Beytotime, Shanghai, China). The density of C666-1 cells was adjusted to 1 × 10^5^ cells/mL. Then, cells were treated with Annexin V and propidium iodide (Beyotime, Shanghai, China) at 4℃ for 15 min without light exposure. The percentage of living, early- or late- apoptotic cells, and necrotic cells were analyzed on the FACSCalibur flow cytometry (BD Biosciences, San Jose, USA).

***Measurement of cell cycle progression*** C666-1 cells were washed twice with cold phosphate buffered saline (Beyotime, Shanghai, China). Next, cells were treated with cell cycle staining kit (MultiSciences Biotech, Hangzhou, China) following the manufacturer’s recommendations. Then, cell cycle distribution was detected by using flow cytometry (BD Biosciences, San Jose, USA).

***Transwell assay*** The invasive potential of C666-1 cells with various treatments was measured using Transwell chamber with 8 μm pore filters (Corning Costar, Corning, USA) as instructed. Matrigel (100 µL; BD Biosciences, San Jose, USA) was used to precoat the upper chamber for 2 h of incubation. C666-1 cells (1 × 10^5^) were cultured in serum-free medium (100 µL) in the upper chambers. In addition, 500 µL Dulbecco’s modified Eagle’s medium including 10% FBS was placed into the bottom chamber. After 48 h of cell incubation, the cells on the lower side of the membrane insert were fixed in 75% methanol for 15 min at room temperature and then stained with 0.1% crystal violet (Sigma-Aldrich, St. Louis, USA) in the dark. The stained cells were imaged using a phase-contrast microscope (Leica, Wetzlar, Germany).

***Wound healing assay*** The treated cells were implanted into 24-well plates and cultured until 95% confluency. The medium was then extracted, and monolayer was perpendicularly scratched using a 10 µL sterile pipette tips. Cell debris was removed through 3 times of PBS washing. After that, cells were put back in culture. The scratched area was imaged at the timepoint of 0 h and 24 h using a microscope (Olympus, Tokyo, Japan) and analyzed by ImageJ software. The gap distance reflects the migratory potential of NPC cells.

***Western blotting*** C666-1 cells with indicated treatment were lysed in radio immunoprecipitation lysis buffer with 10 µL PMSF (Solarbio, Beijing, China). A bicinchoninic acid protein assay kit (Sangon Biotech, Shanghai, China) was used to quantify protein concentration. Then, the protein contents (50 µg) were separated by sodium dodecyl sulfate (SDS)-polyacrylamide gel electrophoresis and transferred to polyvinylidene fluoride membranes (Millipore, Billerica, USA). Next, the membranes were blocked with 5% skim milk for 1 h at ambient temperature followed by incubation with the primary antibodies against AKT (ab8805, 1/500, Abcam, UK), phosphorylated (p)-AKT (ab38449, 1/500, Abcam), P65 (ab32536, 1/1000, Abcam), p-P65 (ab76302, 1/1000, Abcam), IκBα (#9242, 1/1000, Cell signaling technology, Danvers, USA), p-IκB (#2859, 1/1000, Cell signaling technology), Bcl-2 (ab182858, 1/2000, Abcam), Cyclin D1 (ab16663, 1/100, Abcam), p21 (ab109199, 1/1000, Abcam), Bax (ab182733, 1/2000, Abcam) and GAPDH (ab181602, 1/10,000) at 4 °C overnight. Then, the membrane was washed 3 times with Tris buffered saline with Tween-20 and incubated with secondary antibodies for 2 h at ambient temperature. The blots were visualized by enhanced chemiluminescent substrate (Millipore, Billerica, USA) and then quantified by Image J program.

***Statistical analysis*** Data were generated from three independent experiments and analyzed using SPSS22.0 (International Business Machines Corporation, Armonk, USA) and Graphpad prism 8.0 software (GraphPad, La Jolla, USA). Data showed as the mean ± standard deviation. The comparison between two groups was analyzed by Student’s *t* test, and one-way analysis of variance followed by Bonferroni *post hoc* test were applied for comparisons of multi-groups. A value of *p* < 0.05 was defined to be statistically significant.

## Results

### Astaxanthin inhibits C666-1 cell growth, migration, and invasion while promoting cell cycle arrest and apoptosis

**Fig. 1:**
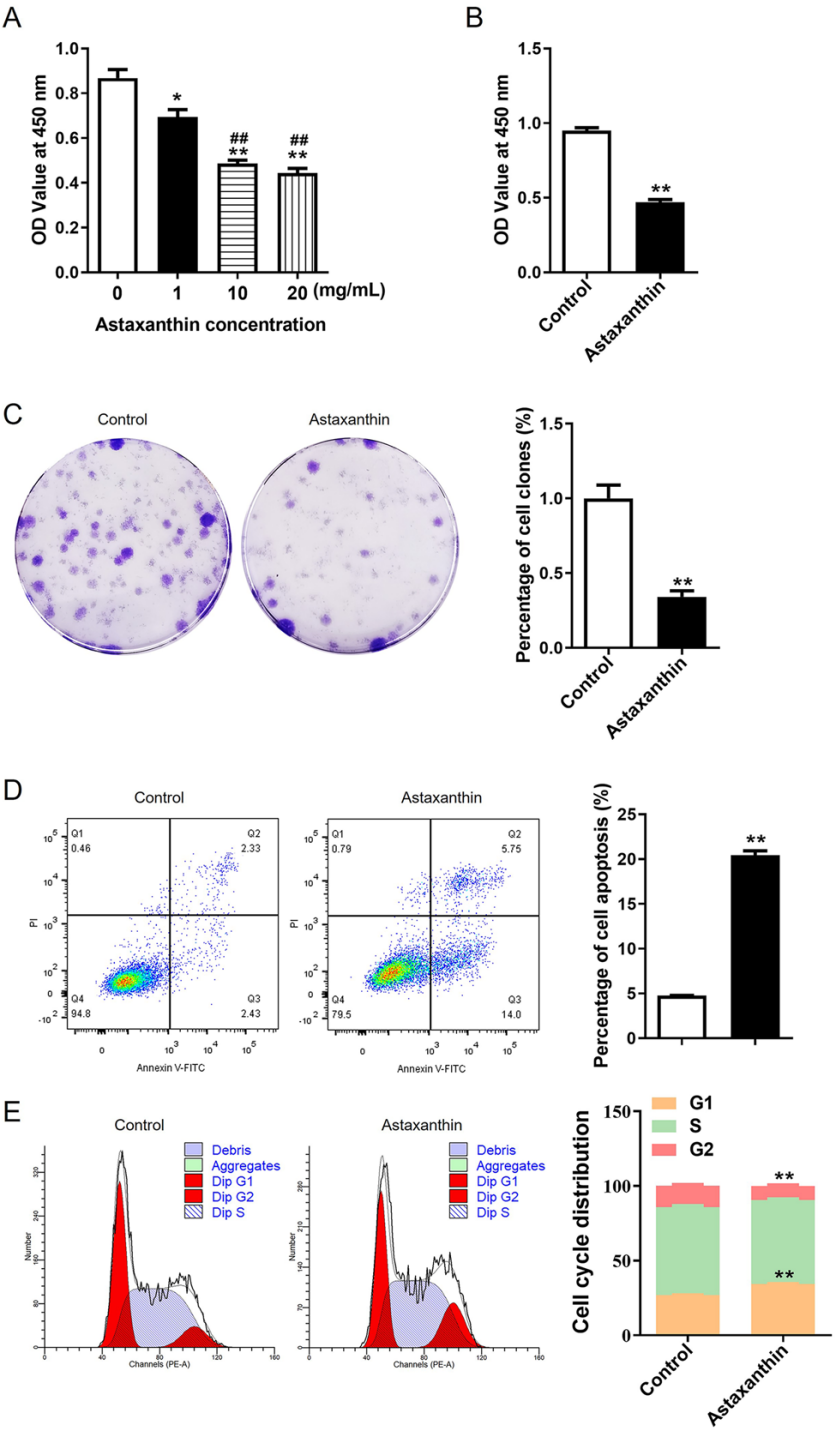
Astaxanthin inhibits C666-1 cell growth while augmenting cell cycle arrest and apoptosis. **A**. The impacts of different concentrations of astaxanthin (1, 10, 20 mg/mL) on viable C66-1 cells were evaluated by CCK-8 assays, and 10 mg/ml astaxanthin was identified for subsequent experiments. **B.** The OD values of C666-1 cells in control and astaxanthin groups were measured by CCK-8 assays. **C**. C666-1 cell proliferation in control or astaxanthin group was measured by colony forming assays. **D-E.** The influence of astaxanthin on C666-1 cell apoptotic rate (D) and cell cycle progression (E) were detected using flow cytometry. ^*^p < 0.05, ^**^p < 0.01, ^##^*p* < 0.01

**Fig. 2:**
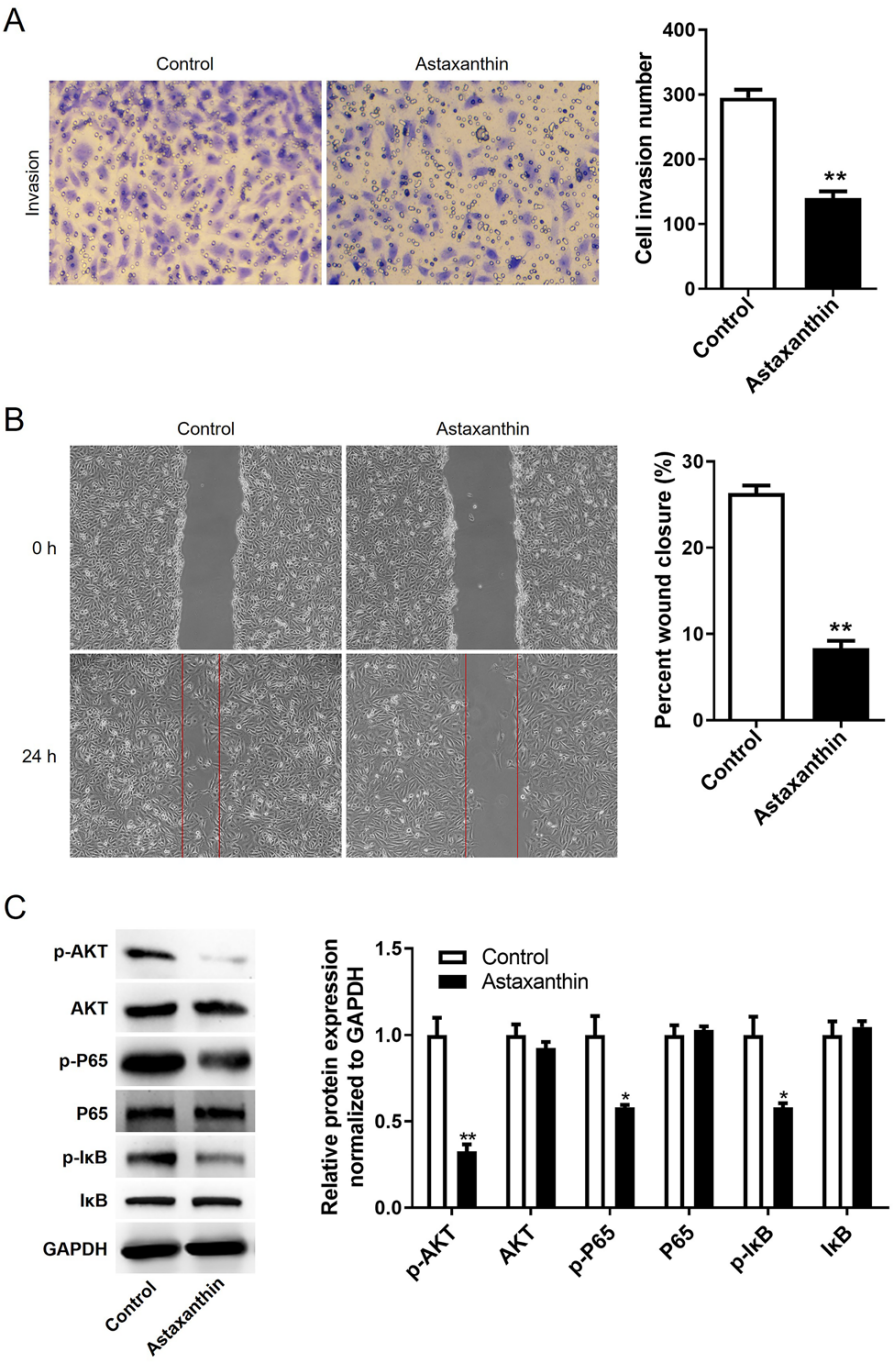
Astaxanthin hampers C666-1 cell migration and invasion and suppresses the activation of PI3K/AKT and NF-κB pathways. **A-B**. Transwell and wound healing assays were performed to measure the number of invaded cells and the migration of C666-1 cells after astaxanthin treatment. **C**. The protein levels of factors involved in PI3K/AKT pathway (p-AKT, AKT) and NF-κB pathway (p-P65, p-IκB, P65, and IκB) were quantified using western blotting. ^*^p < 0.05, ^**^p < 0.01

CCK-8 assays were performed to measure C666-1 cell viability in response to different concentrations of astaxanthin (1, 10, 20 mg/mL). As shown by Fig.  1 A, C666-1 cell viability was inhibited by astaxanthin in a dose-dependent manner. In addition, 10 mg/mL astaxanthin reduced cell viability to nearly 50% (Fig. 1^1^A-1B). Thus, 10 mg/mL astaxanthin was utilized for subsequent experiments. The number of formed cell colonies was also decreased by astaxanthin compared to that of the control group (Fig. 1 C). The results indicated the suppressive impact of astaxanthin on NPC cell proliferation. The data from Fig. 1D revealed that the percentage of apoptotic cells was obviously increased after astaxanthin stimulation. It was also found that the C666-1 cells with astaxanthin stimulation were blocked in G1 stage, as demonstrated by a significant increase in cell population at G1 phase and a decrease in cell number at G2 phases compared with those in the control group (Fig. 1E). The number of invaded cells and the percentage of wound closure rate were significantly reduced in the context of astaxanthin, according to results of wound healing assay and Transwell assay (Fig. 2A and B). These findings demonstrated that the migratory and invasive abilities of C666-1 cells were suppressed by astaxanthin stimulation. Overall, astaxanthin effectively dampens C666-1 cell proliferation, metastasis, and invasion while enhancing cell cycle arrest and apoptosis.

### Astaxanthin inhibits the activity of PI3K/AKT and NF-κB signaling

It is well documented that PI3K/AKT and NF-κB pathways are recognized as crucial players in tumor initiation and development, including NPC [25, 26]. Moreover, astaxanthin was reported to exert an anti-tumor role in various cancers, such as oral cancer and hepatocellular carcinoma, by inhibiting PI3K/AKT and NF-κB signalling [27]. Western blotting was conducted to quantify protein levels of factors involved in two signaling pathways. As displayed in Fig. 2C, phosphorylated levels of AKT, P65 and IκBα were markedly reduced by astaxanthin treatment, while the protein levels of AKT, P65 and IκB had no significant changes. These results indicated that astaxanthin may inhibit malignant behaviors of C666-1 cells by blocking PI3K/AKT and NF-κB signalling.

### Astaxanthin upregulates miR-29a-3p expression to inactivate the PI3K/AKT and NF-κB pathways

**Fig. 3:**
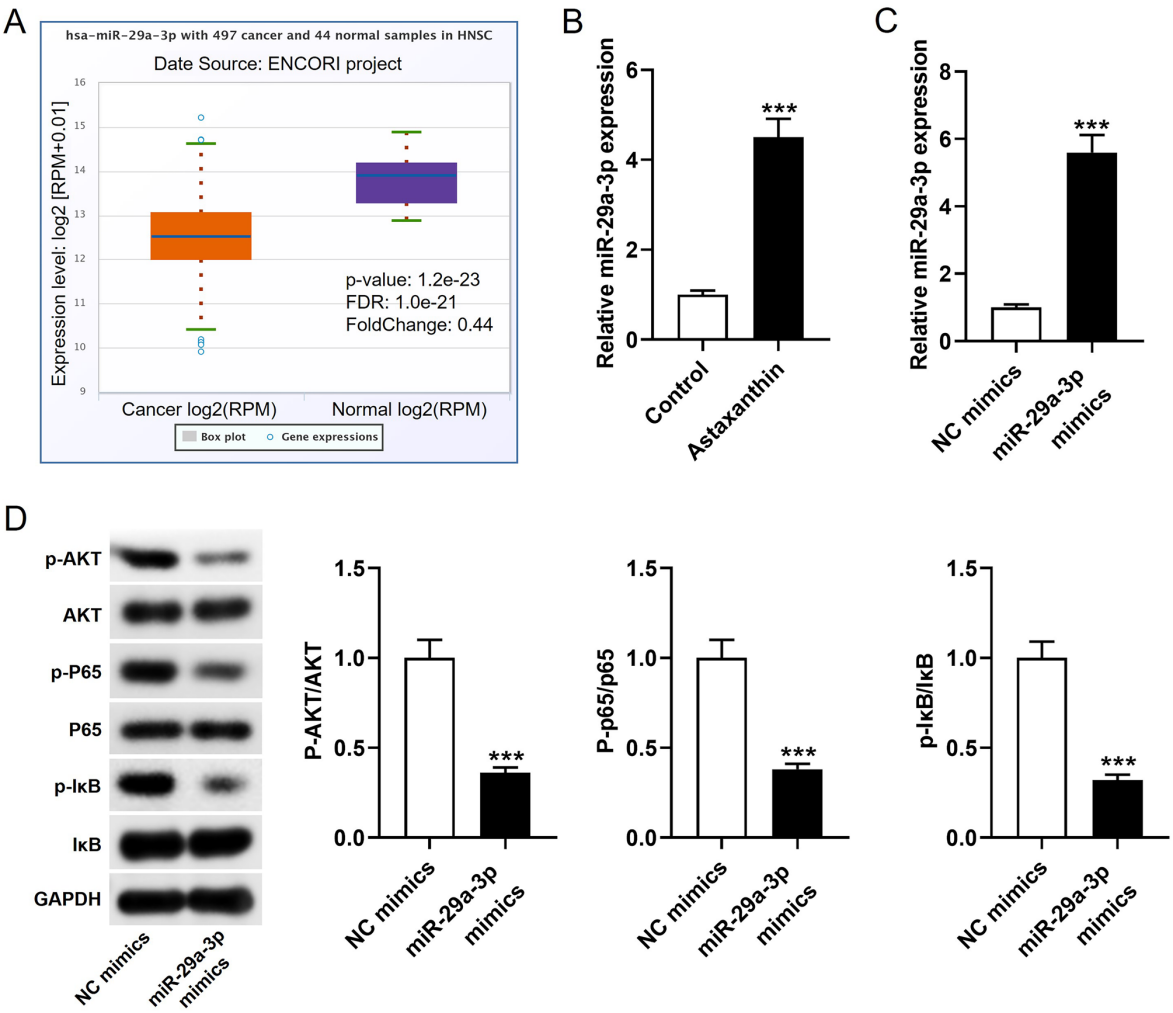
Astaxanthin upregulates miR-29a-3p expression to inactivate the PI3K/AKT and NF-κB pathways.**A.** miR-29a-3p expression in head and neck squamous cell carcinoma (HNSC) samples and normal samples was analyzed by the tool ENCORI, encyclopedia of RNA interactomes. **B**. miR-29a-3p expression in C666-1 cells treated with or without astaxanthin was measured by RT-qPCR. **C**. After transfection of mimics into C666-1 cells, RT-qPCR was performed to assess miR-29a-3p expression in miR-29a-3p mimics or NC mimics groups. **D**. Western blotting was conducted to measure protein levels of factors associated with PI3K/AKT or NF-κB signaling pathways in C666-1 cells transfected with miR-29a-3p mimics or NC mimics. ***p<0.001.

Astaxanthin was previously reported to increase miR-29a-3p expression in colorectal cancer and cardiovascular diseases [15, 28]. ENCORI, encyclopedia of RNA interactomes, shows that miR-29a-3p is downregulated in head and neck squamous cell carcinoma (HNSC) (Fig. 3A). MiR-29a-3p is also closely associated with PI3K and NF-κB signaling pathways during cancer development [29, 30]. Therefore, subsequent experiments were conducted to explore whether miR-29a-3p participates the regulatory network mediated by astaxanthin. Results of RT-qPCR revealed that miR-29a-3p level was upregulated by astaxanthin in NPC cells (Fig. 3B). To explore the effect of miR-29a-3p on PI3K and NF-κB signaling pathways, miR-29a-3p mimics or negative control (NC) mimics were transfected into NPC cells for 48 h to overexpress miR-29a-3p. RT-qPCR revealed that miR-29a-3p expression was successfully amplified in the miR-29a-3p mimics group relative to that in NC mimics group (Fig. 3C). Results of western blotting elucidated that the phosphorylated levels of AKT, p65, and IκB were prominently reduced in response to miR-29a-3p (Fig. 3D), implying the suppressive effect of miR-29a-3p on PI3K/Akt and NF-κB pathways.

### Astaxanthin suppresses malignant behaviors of C666-1 cells by inhibiting PI3K/AKT and NF-κB signaling

**Fig. 4:**
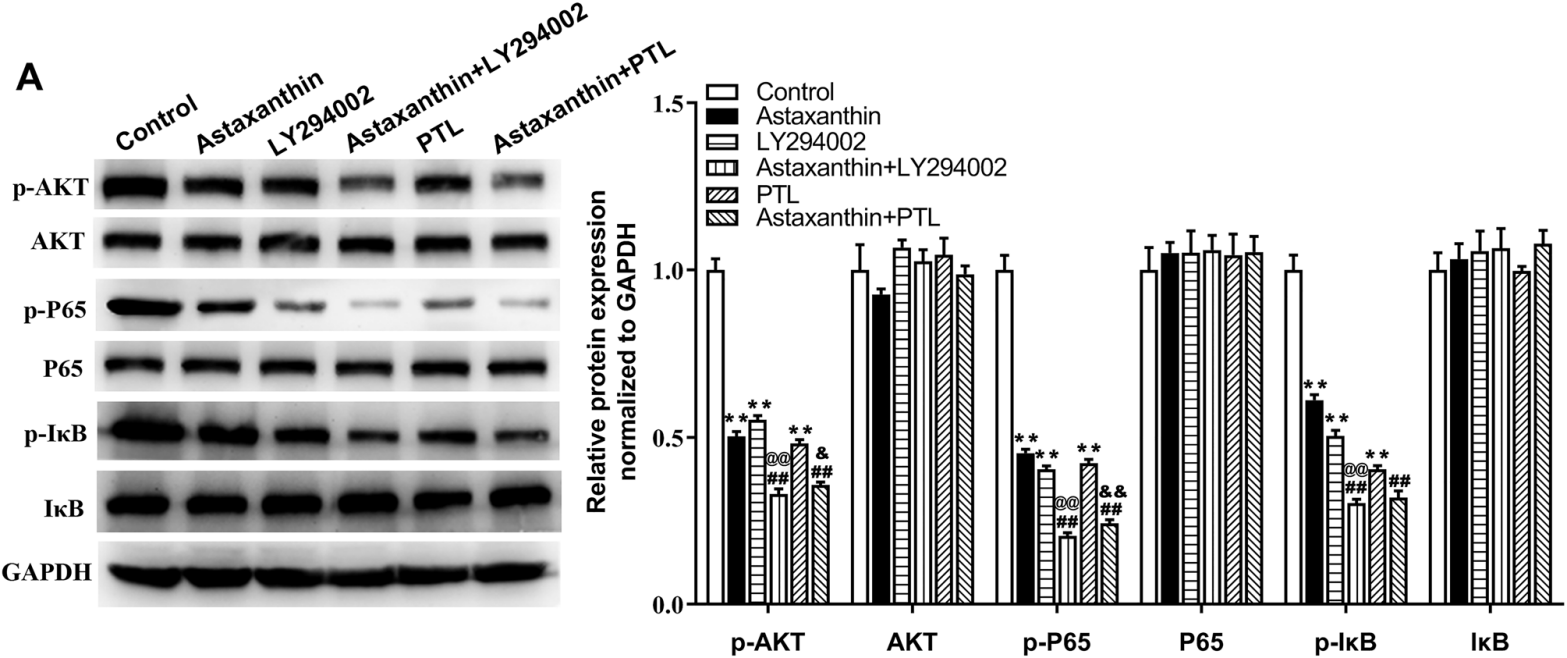
Astaxanthin inhibits the activation of PI3K/AKT and NF-κB signaling. **A**. After astaxanthin, LY294002 or PTL treatment, the protein levels of factors involved in PI3K/AKT pathway (p-AKT, AKT) and NF-κB pathway (p-P65, p-IκB, P65, and IκB) were quantified by western blotting. ^**^p < 0.01 vs. control; ^##^*p* < 0.01 vs. astaxanthin; ^@@^*p* < 0.01 vs. LY294002, ^&^*p* < 0.05, ^&&^*p* < 0.01 vs. PTL

**Fig. 5:**
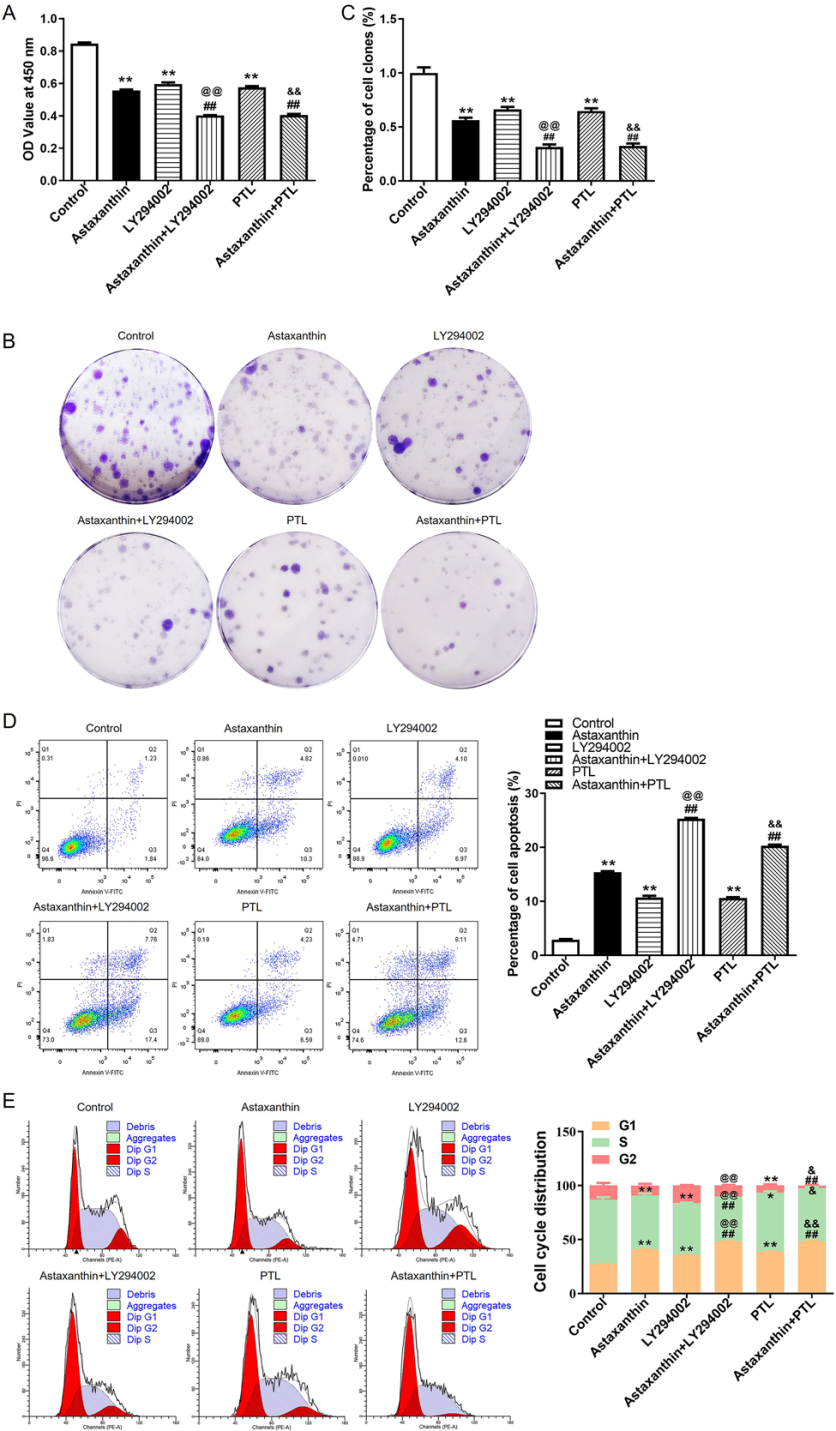
Astaxanthin suppresses C666-1 cell growth by inhibiting PI3K/AKT and NF-κB signaling. A-E. After astaxanthin, LY294002 or PTL treatment, C666-1 cell viability **(A)**, proliferation **(B-C)**, apoptosis **(D)** and cell cycle progression **(E)** were evaluated using CCK-8 assays (A), colony forming assays **(B-C)**, and flow cytometry analysis (D-E).^*^p < 0.05, ^**^p < 0.01 vs. control; ^##^*p* < 0.01 vs. astaxanthin; ^@@^*p* < 0.01 vs. LY294002, ^&^*p* < 0.05, ^&&^*p* < 0.01 vs. PTL

**Fig. 6:**
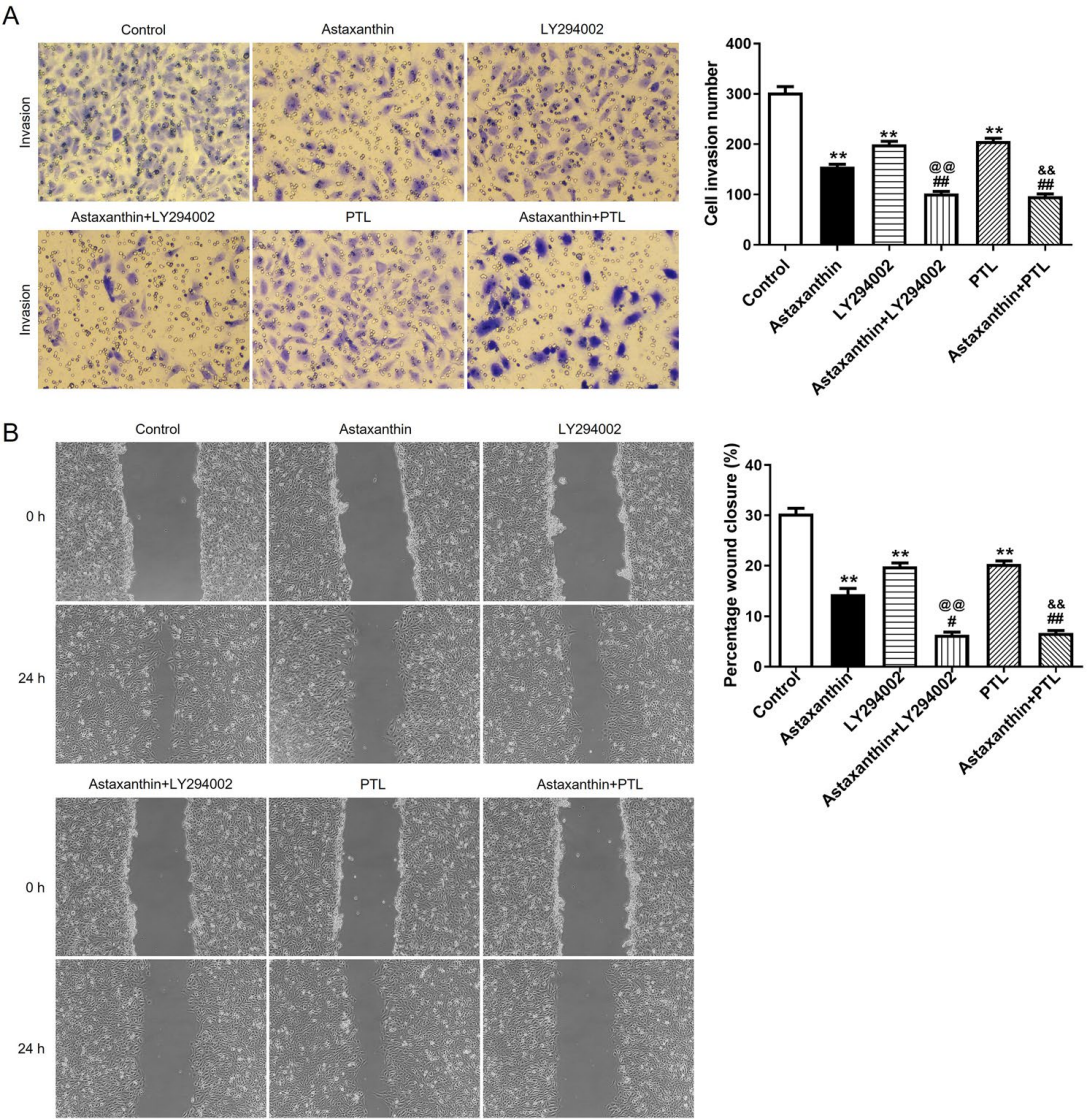
Astaxanthin suppresses C666-1 cell migration and invasion by inhibiting PI3K/AKT and NF-κB signaling. **A-B**. After astaxanthin, LY294002 or PTL treatment, C666-1 cell invasion **(A)** and migration **(B)** was evaluated using Transwell and wound healing assays. ^**^p < 0.01 vs. control, ^##^*p* < 0.01 vs. astaxanthin, ^@@^*p* < 0.01 vs. LY294002, ^&&^*p* < 0.01 vs. PTL

To further validate whether astaxanthin obstructs malignant behavior of C666-1 cells by inactivating the two pathways, LY294002 (PI3K/AKT inhibitor) or PTL (NF-κB inhibitor) was applied to block the two pathways. As displayed in Fig. 4A, the p-AKT level was reduced after LY294002 treatment, and this effect was amplified by astaxanthin + LY294002. Similarly, the p-P65 and p-IκB levels were reduced after PTL treatment, and the alteration was promoted by astaxanthin stimulation. Data from CCK-8 assays suggested that the OD values of C666-1 cells were reduced after astaxanthin, LY294002 or PTL treatment (**p < 0.01), and these phenomena were strengthened when LY294002 or PTL combined with astaxanthin ( ^@@^*p* < 0.01, ^&&^*p* < 0.01) (Fig. 5A). The reduction of cell colonies caused by LY294002 or PTL was strengthened by astaxanthin stimulation (Fig. 5B-C). After the blockage of PI3K/AKT or NF-κB pathway, C666-1 cell apoptotic rate was increased, and astaxanthin stimulation enhanced thepro-apoptosis impact exerted by LY294002 or PTL (Fig. 5D). Figure 5E revealed that LY294002 or PTL induced G1 phase arrest compared with that in the control, and the cell cycle arrest was strengthened by astaxanthin treatment (Fig. 5E). The data from Transwell and wound healing assays implied that the decline in the number of invaded and migrated C666-1 cells induced by LY294002 or PTL was aggravated by astaxanthin stimulation (Fig. 6A-B). All these results indicated that blocking PI3K/AKT or NF-κB signalling enhanced the inhibitory effect of astaxanthin on C666-1 cell proliferation, migration, and invasion and amplified the promotion of astaxanthin on cell apoptosis and G1 phase arrest.

### Astaxanthin alters protein levels of cell cycle markers and apoptotic markers by inactivating PI3K/AKT or NF-kappaB signaling

**Fig. 7:**
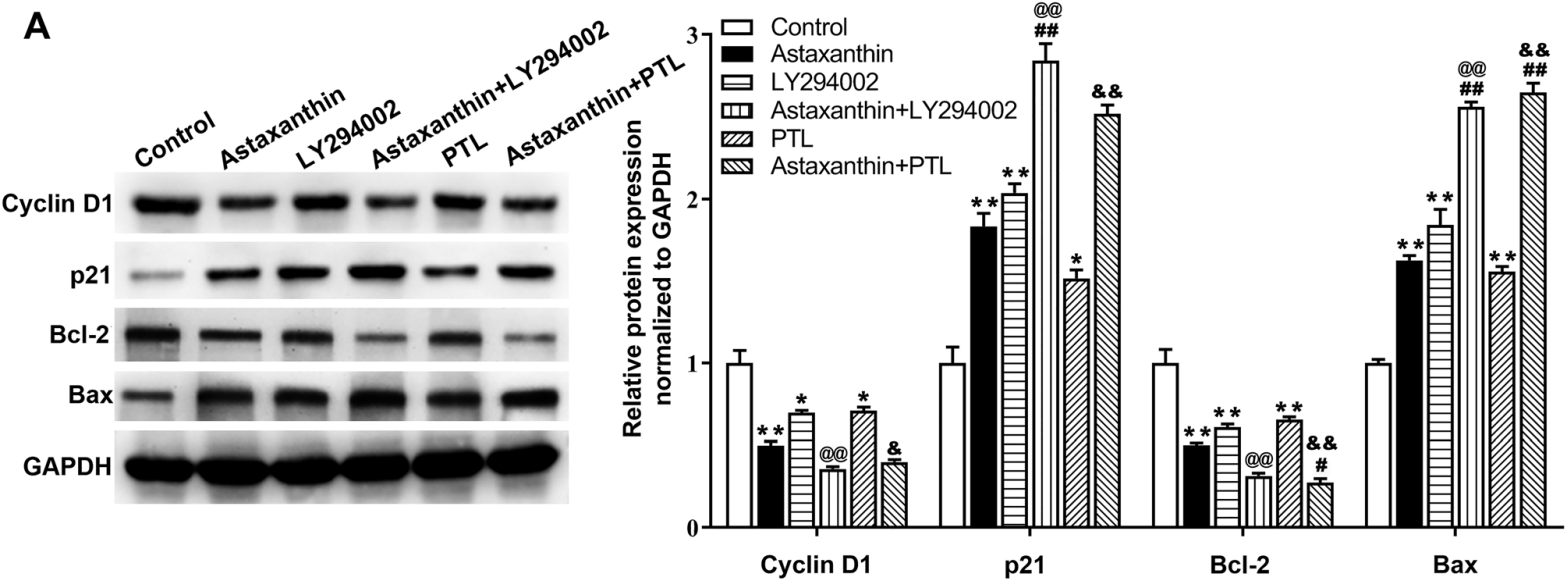
Astaxanthin alters protein levels of cell cycle markers and apoptotic markers by inactivating PI3K/AKT or NF-kappaB signaling. **A.** Protein expression of cell cycle markers (cyclin D1 and p21) and apoptotic markers (Bcl-2 and Bax) in response to LY294002 or PTL treatment were quantified using western blotting. ^**^p < 0.01 vs. control, ^##^*p* < 0.01 vs. astaxanthin, ^@@^*p* < 0.01 vs. LY294002, ^&^*p* < 0.05, ^&&^*p* < 0.01 vs. PTL

The protein expression of cell cycle markers (Cyclin D1 and p21) and cell apoptosis markers (Bcl-2 and Bax) were quantified after astaxanthin, LY294002 or PTL treatment. The data from Fig. 7A displayed that astaxanthin reduced CyclinD1 and Bcl-2 levels while increasing p21 and Bax levels. LY294002 or PTL treatment also led to the decrease in CyclinD1 and Bcl-2 as well as the increase in p21 and Bax levels (Fig. 7A). Importantly, after the co-treatment of LY294002 + astaxanthin or PTL + astaxanthin, CyclinD1 and Bcl-2 protein levels were more obviously weakened compared with their levels in the single group of LY294002, PTL or astaxanthin, while p21 and Bax protein expression were obviously higher than that in the cell group with single treatment. Hence, our results suggested that astaxanthin can alter the expression of apoptotic markers and cell cycle markers by blocking the PI3K/AKT and NF-κB signaling, thereby inhibiting the malignant behaviors of C666-1 cells.

## Discussion

Chemoprevention refers to the usage of natural, biological, or synthetic agents to prevent or inhibit tumorigenesis [31]. Among which, the application of natural agents may be an ideal option for tumor treatment because nature agents abundant, at low cost, and have less toxicity. More and more studies were designed to explore the anti-tumor impact of natural extracts. For example, β-Elemene nitric oxide derivatives, the extraction from the rhizome of Curcuma *wenyujin*, was reported to exert the antitumor efficacy in malignant brain glioma both in vitro and in vivo [32]. Demethylzeylasteral was extracted from Tripterygium wilfordii Hook. f. and was verified to dampen the tumorigenicity of liver cancer stem cells by targeting lactate to suppress histone lactylation [33]. The anti-cancer role of astaxanthin has recently attracted attention. As reported, astaxanthin suppresses carcinogenesis of breast, colon, prostate, oral cavity, and urinary bladder [11]. However, the functions of astaxanthin in NPC and the potential mechanism have never been revealed.

NPC, a highly invaded malignant cancer, often had invaded adjacent regions and even metastasized to distant organs when diagnosed [34]. Tumor cell proliferation and differentiation are associated with tumor invasion, and thus the suppression of malignant NPC cell process is important in cancer therapy [35]. Astaxanthin, in our work, was found to inhibit C666-1 cell growth, arrest C666-1 cell cycle, induce C666-1 cell apoptosis and block C666-1 cell mobility.

Recently, astaxanthin has been reported to increase the expression of miR-29a-3p and miR-200a in colorectal cancer, thereby targeting MMP2 and ZEB1 and eventually inhibiting the epithelial-mesenchymal transition of cancer cells [15]. The upregulation of miR-29a-3p is also mentioned in cardiovascular diseases [28]. In NPC, miR-29a-3p is known as a tumor suppressor and can improve radiotherapy sensitivity of NPC cells by targeting COL1A1 [36]. The present study verified that astaxanthin upregulated miR-29a-3p expression in C666-1 cells, which is not aberrant from findings of previous studies [15, 28]. In addition, the present study showed that overexpression of miR-29a-3p inhibited the activity of PI3K/AKT and NF-κB signaling. The influence of miR-29a-3p on the activity of PI3K/AKT and NF-κB signaling in NPC has not been reported yet. However, miR-29a-3p is shown to suppress the PI3K/AKT or NF-κB signaling in other types of cancer such as glioma [37] and gastric cancer [38].

The occurrence of NPC is accompanied with the dysregulated crucial hallmarks and the abnormal activation of significant pathways, such as PI3K/AKT and NF-κB [34]. PI3K/AKT pathway is a classical one for the regulation of cell proliferation, apoptosis, metastasis, and gene transcription [39]. NF-κB is overexpressed in nearly all NPC tissues according to previous literature [39]. Blocking NF-κB activation strengthened the apoptotic response, reduced growth and clonogenic survival in several human cancer cells, including NPC cells [25]. Moreover, a recent study from Kowshik Jaganathan et al. reported that astaxanthin can inhibit oral cancer cells behaviors by abrogating PI3K/AKT, NF-κB, and STAT3 pathway [13]. In the article written by Kowshik Jaganathan et al., the effects of astaxanthin on the proliferation and apoptosis of human oral squamous carcinoma cells (SCC-4) and rat squamous cell carcinoma cell (SCC-131) was explored, and PI3K inhibitor Wortmannin as well as NF-κB inhibitor Bay-11 were used to investigate the relationship between astaxanthin and the two signaling pathways in oral cancer [13]. Consistent with the article, the present study used LY294002 (PI3K/AKT inhibitor) and/or PTL (NF-κB inhibitor) to conduct experiments. We discovered that LY294002 and/or PTL strengthened the inhibitory effect of astaxanthin on malignant cell behavior, suggesting that the suppressive impact of astaxanthin on C666-1 cell growth and mobility was realized partly by inactivating PI3K/AKT and/or NF-κB pathway. In addition, in the reference written by Kowshik Jaganathan et al., a hamster model of oral carcinogenesis was established to further validate the conclusions of in vitro experiments. In this study, animal experiments were not conducted for in vivo verification, which is a limitation of the current study.

Dysregulated Cyclin D1 is an oncogene and is regarded as a driver of solid tumors [40]. Previous studies found that Cyclin D1 was significantly upregulated in NPC and positively related to its progression [41, 42]. Cyclin D1 can be the downstream regulatory factor of NF-kappaB p65 [43]. P21 is a cyclin-dependent kinase inhibitor, also named CDKN1A, which is activated by p53 at the transcriptional level and serves as a tumor suppressor [44]. Moreover, a low p21 level results in poor prognosis of patients with NPC [45]. Bcl-2 acts as an influential factor in favoring cell survival by suppressing cell apoptosis [46]. Moreover, Bcl-2 has been discovered to promote NPC invasion, growth and metastasis [47]. Bax is a X protein associated with Bcl-2 and is responsible for mitochondrial-regulated cell death by permeabilizing the outer mitochondrial membrane [48]. After the permeabilization of Bax, cytochrome C can be released into the cytosol, leading to caspase activation and eventually cell apoptosis [49]. Moreover, Bcl-2 and Bax have been reported to be related to NPC progression [50]. The results of our study revealed that astaxanthin inhibited Cyclin D1 and Bcl-2 protein expression and elevated p21 and Bax protein expression, and the alterations were strengthened by LY294002 or PTL. These results revealed that astaxanthin might exert a repressive impact on C666-1 cell process by affecting proteins related to cell proliferation, apoptosis, and cycling.

Although some meaningful results have been achieved from our study, the limitations in the research should be revealed. First, our experiments were performed using C666-1 cells, and the results were not validated in another NPC cell line or animal models. Second, it is still unknown that whether astaxanthin directly or indirectly exerts its anti-cancer effect through the PI3K/AKT and NF-κB pathways. The third point is that the effect of astaxanthin on other biological processes such as calcium homeostasis and hematotoxicity were not further investigated.

In conclusion, it was verified that astaxanthin inhibits C666-1 cell proliferation, arrests cell cycle progression, induces apoptosis and blocks cell mobility in vitro by inactivating PI3K/AKT and NF-κB pathways. Our study first reveals the anticancer role of astaxanthin in NPC and illustrates that astaxanthin has the potential to be a promising therapeutic agent for human NPC.

## Data Availability

The datasets generated during and/or analysed during the current study are available from the corresponding author on reasonable request.
